# Comparative analysis of surgical prognostic between HIC and NHIC patients after cystoscopy with hydrodistention

**DOI:** 10.1097/MD.0000000000039640

**Published:** 2024-09-20

**Authors:** Lin Zhu, Hanwei Ke, Qi Wang, Kexin Xu

**Affiliations:** a Department of Dermatology, Sexually Transmitted Diseases, and Medical Aesthetics, Affiliated Beijing Chaoyang Hospital of Capital Medical University, Beijing, China; b Department of Urology, Peking University People’s Hospital, Beijing, China; c Peking University Applied Lithotripsy Institute, Peking University People’s Hospital, Beijing, China.

**Keywords:** clinical features, cystoscopy with hydrodistention, Hunner lesion interstitial cystitis, interstitial cystitis, non-Hunner lesion interstitial cystitis, prognostic

## Abstract

This study aims to clarify the pathogenic mechanism of interstitial cystitis (IC), which has led to uncertainty in its diagnosis and treatment. We examined data from 18 interstitial cystitis with Hunner lesions (HIC) and 18 interstitial cystitis without Hunner lesions (NHIC) patients, including their clinical information, urodynamic test results, and maximum bladder capacity. A 1-year follow-up tracked disease progression. Postoperative recovery showed that HIC patients experienced significantly greater improvements in Visual Analog Scale pain scores compared to NHIC patients (*P* = .0049). This trend continued at the 6-month mark (*P* = .0056). Over the 1-year follow-up, NHIC patients exhibited a statistically significant improvement in Pain and Urgency/Frequency scores, while HIC patients had a gradual overall score increase from preoperative to postoperative stages. However, no significant differences were observed in either group at 1 year postoperatively compared to preoperative scores. This study revealed distinct differences between HIC and NHIC patients, including reduced bladder volumes and more severe nociceptive pain in HIC patients. Early analgesic interventions effectively alleviated discomfort in HIC patients. The combination of cystoscopic hydrodistention and water dilatation was highly effective in relieving pain symptoms in HIC patients but increased the risk of recurrence, necessitating recurrent bladder infusion and timely therapeutic adjustments. In contradiction to prior paradigms, the surgical intervention of cystoscopic water hydrodistention also yielded favorable outcomes among NHIC patients.

## 1. Introduction

Hunner^[1]^ delineated a condition of bladder ulcer, characterized by vivid crimson mucosal patches and intricate vasculature, giving rise to pale cicatrices converging toward the center. This manifestation was accompanied by a sense of urinary urgency and discomfort in the bladder area.^[1]^ Fall et al^[2]^ introduced the classification of bladder pain syndrome/interstitial cystitis (BPS/IC), dividing it into 2 distinct entities: Hunner lesion interstitial cystitis (HIC) and non-HIC (NHIC). They expounded upon the discernible histological disparities between these 2 subtypes, emphasizing the necessity of studying them independently to gain deeper insights into their unique characteristics.^[2]^

Previous investigations have established that patients diagnosed with HIC commonly present with an advanced age at the time of diagnosis, in contrast to NHIC patients. Furthermore, HIC patients exhibit many distinct histological features, including epithelial denudation and chronic inflammatory modifications characterized by lymphoplasmacytic and hypertrophic infiltrates, interstitial fibrosis, and edema.^[3,4]^ In contrast, NHIC patients do not have such histological features.^[5]^ NHIC and HIC demonstrate pronounced disparities in their phenotypic profiles. However, it is noteworthy that these 2 subtypes are not differentiated in the current clinical diagnostic and therapeutic practices across many regions.

For clinical diagnosis, the identification and categorization of HIC and NHIC can only be accomplished by means of cystoscopy with hydrodistention (CWH), however, the utilization of this procedure in routine clinical practice is not yet commonplace. Notably, the 2022 American Urological Association guidelines for BPS/IC endorse the recommendation that patients with suspected HIC should undergo CWH as part of the diagnostic process,^[6]^ this statement has engendered contentious discussions among clinical experts who participated in the formulation of prior guidelines.^[7]^ Our research group maintains the standpoint that discerning between distinct subtypes is imperative, given its substantial influence on disease prognosis. Accordingly, the present study endeavors to elucidate disparities in the postoperative prognosis of patients presenting with both subtypes of BPS/IC subsequent to the initial CWH intervention.

## 2. Materials and methods

### 2.1. Ethics approval and informed consent

This study was approved by the Ethics Committee of the Peking University People’s Hospital. Data were processed in accordance with the Declaration of Helsinki. Informed consent was obtained from all participants.

### 2.2. Patients

Between January 2018 and July 2021, we did a prospective cohort study comprising a total of 43 patients underwent the CWH procedure (under spinal or general anesthesia, utilizing a rigid cystoscope to maintain bladder instillation at a pressure of 80 cmH_2_O for a duration of 3 to 5 minutes. Document the presence of bladder diverticula/trigones, mucosal alterations during bladder filling and voiding (including glomerulation resembling hemorrhage), and the extent of Hunner lesions (defined as radiating pale scars surrounded by small vessels) during cystoscopy. In order to ensure the homogeneity of the study population, strict inclusion and exclusion criteria were employed. The inclusion criteria encompassed female patients who received a diagnosis of BPS/IC and subsequently underwent CWH surgery at Peking University People’s Hospital within the specified timeframe. Moreover, patients were required to undergo regular outpatient or telephone follow-up for a minimum duration of half a year following the surgical intervention. Exclusion criteria were established to exclude patients with a history of urinary system tumors, positive urine culture, neurogenic bladder, eosinophilic cystitis, or ketamine-related cystitis. Additionally, patients who did not complete the intended postoperative outpatient or telephone follow-up were excluded from the study cohort. Consequently, a group of 36 patients fulfilled the aforementioned inclusion criteria and constituted the study sample.

### 2.3. Clinical data

During hospitalization, we documented the general clinical data, urodynamic examination results, and maximum bladder capacity under anesthesia for these 36 patients. The general clinical data encompassed variables such as age, number of hydrodistention sessions, number of bladder perfusion, as well as the specific medications employed for perfusion. To comprehensively assess the patients’ symptoms and overall experience, we distributed standardized questionnaires during their hospital stay. These questionnaires included the employment of the Visual Analog Scale (VAS) for pain assessment, the Interstitial Cystitis Problem Index (ICPI), the Interstitial Cystitis Symptom Index (ICSI), the Pelvic Pain and Urgency/Frequency (PUF) Symptom Scale, and the Overactive Bladder Symptom Score (OABSS). Furthermore, we recorded the maximum bladder capacity under anesthesia during the surgical procedure. The urodynamic data encompassed variables such as maximum bladder capacity, bladder compliance, detrusor pressure at maximum flow rate, maximum detrusor pressure, and residual urine volume.

### 2.4. Cystoscopy with hydrodistension

Under spinal anesthesia, a rigid cystoscope was employed to instill the bladder with normal saline from a height of 80 cm for 3 to 5 minutes. During bladder filling and emptying, the presence of bladder diverticula/trabeculae, mucosal changes (glomerulations), and Hunner lesions (characterized as reddened mucosal plaques with small blood vessels radiating toward a central pale scar) were documented. The grading of bladder mucosal hemorrhage was categorized into 5 levels: grade 0 for normal mucosa; grade 1 for petechiae in at least 2 quadrants; grade 2 for extensive submucosal bleeding; grade 3 for diffuse global mucosal bleeding; and grade 4 for mucosal disruption, including Hunner lesions.^[7]^

### 2.5. Statistical analyses

Data processing and analysis were conducted using R software (version 3.6.3). Descriptive statistics were employed to summarize the continuous variables with a normal distribution, presenting them as mean ± standard deviation. For variables that did not conform to a normal distribution, the median and interquartile range were utilized. To compare 2 independent samples with normally distributed variables, the independent samples *t* test was employed. In the case of nonnormally distributed data, the Wilcoxon rank sum test was utilized for statistical analysis. Furthermore, for nonnormally distributed data involving multiple group comparisons, the Kruskal–Wallis rank sum test was employed, with Bonferroni correction utilized for multiple comparisons. Statistical significance was denoted as *P < .05, **P < .005, and ***P < .001.

## 3. Results

**Figure 1.**
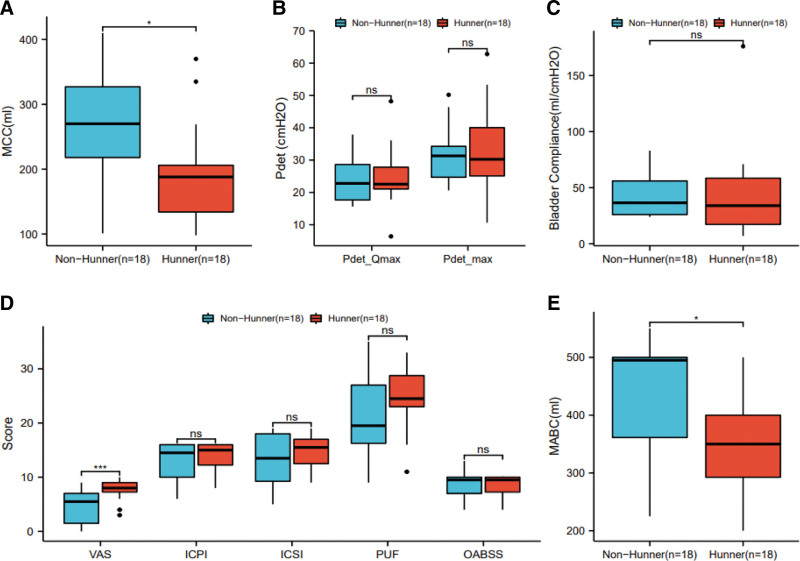
Comparative analysis of clinical parameters between HIC and NHIC patient cohorts. (A) Comparison of urinary flow rate (mL/s) between HIC and NHIC patient cohorts. (B) Comparison of peak urinary flow rate (Qmax, mL/s) between HIC and NHIC patient cohorts. (C) Comparison of voiding time (s) between HIC and NHIC patient cohorts. (D) Comparative scores across various scales for HIC and NHIC patient cohorts, including VAS, ICSI, ICPI, PUF Symptom Score, and O’Leary-Sant Interstitial Cystitis Symptom Index (OABSS). (E) Comparative postoperative O’Leary-Sant Problem Index (OABSS) scores between HIC and NHIC patient cohorts. HIC = interstitial cystitis with Hunner lesions, ICPI = Interstitial Cystitis Problem Index, ICSI = Interstitial Cystitis Symptom Index, NHIC = interstitial cystitis without Hunner lesions, NS = not significant (*P* > .05), OABSS = Overactive Bladder Symptom Score, PUF = Pelvic Pain and Urgency/Frequency, VAS = Visual Analog Scale. *P < .05. ***P < .001.

As depicted in Table 1, the cohort of this study comprised 18 NHIC patients and 18 HIC patients. HIC patients exhibited a significantly lower maximum bladder capacity under anesthesia (350 [292.5–400] vs 495 [361.25–500], *P* = .013) and urodynamic maximum bladder capacity ([192.35 ± 77.06] vs [265.12 ± 92.83], *P* = .018) compared to NHIC patients. There was no significant difference in other indicators (Fig. 1).

**Table 1:** Basic demographic data of NHIC and HIC patients.

Clinical data	NHIC	HIC	*P*
N	18	18	
Age	52.5 (33.25–63.75)	62 (35.75–70.75)	.121
Follow-up time (mo)	17.5 (7–28.75)	8 (6–17)	.058
Number of hydrodistension procedures, n (%)			.692
1	14 (77.7%)	15 (83.3%)	
2	1 (5.6%)	2 (11.1%)	
≥3	3 (16.7%)	1 (5.6%)	
Bladder perfusion medication, n (%)			.432
Not perfused	2 (11.1%)	0 (0%)	
Quadruple therapy	6 (33.3%)	4 (22.2%)	
Cystistat	8 (44.5%)	12 (66.6%)	
Cystistat + quadruple therapy	2 (11.1%)	2 (11.2%)	
Number of instillations	16 (8–20)	12 (9.25–19.75)	.766
Preoperative ICPI score	14.5 (10–16)	15 (12.25–16)	.360
Preoperative ICSI score	13.5 (9.25–18)	15.5 (12.5–17)	.555
Preoperative PUF score	20.56 ± 7.16	24.28 ± 5.38	.087
Preoperative OABSS score	9.5 (7–10)	9.5 (7.25–10)	.765
Anesthesia-induced maximum bladder capacity (mL)	495 (361.25–500)	350 (292.5–400)	.013*
Maximum bladder capacity (mL)	265.12 ± 92.83	192.35 ± 77.06	.018*
PdetQmax (cmH_2_O)	24.12 ± 7.24	24.92 ± 9.83	.804
Pdet (cmH_2_O)	32.05 ± 8.87	32.94 ± 13.64	.836
Bladder compliance (mL/cmH_2_O)	36.5 (26–56)	34 (17.25–58.5)	.394
RUV(mL)	0 (0–53.5)	28.5 (2.5–53.25)	.293

ICPI = Interstitial Cystitis Problem Index, ICSI = Interstitial Cystitis Symptom Index, OABSS = Overactive Bladder Syndrome Score, Pdet = maximum detrusor pressure, PdetQmax = maximal detrusor pressure at maximal flow rate, PUF = Pelvic Pain and Urgency/Frequency, RUV = residual urine volume.

* *P* < .05.

We performed an analysis of the changes in preoperative scores and postoperative scores at 1 and 6 months based on the follow-up data. By comparing the corresponding indicators before and after the surgery for each patient, we evaluated the symptomatic improvement in NHIC and HIC patients during the 6-month postoperative period. Compared to preoperative values, NHIC patients exhibited a significant reduction in VAS scores, ICSI scores, ICPI scores, PUF scores, and OABSS scores at 1 and 6 months post-surgery (*P* < .05), as did HIC patients (*P* < .05) (Supplementary Figures 1–5, Supplemental Digital Content, http://links.lww.com/MD/N585, http://links.lww.com/MD/N586, http://links.lww.com/MD/N587, http://links.lww.com/MD/N588, http://links.lww.com/MD/N589).

Based on the analysis of postoperative improvement compared to preoperative scores, Supplementary Figure 6A, Supplemental Digital Content, http://links.lww.com/MD/N590, shows that HIC patients had a more significant improvement in VAS pain scores at 1 month postoperatively compared to NHIC patients, with a *P* value of .0049. In terms of recovery at 6 months postoperatively compared to preoperative scores, HIC patients still exhibited a more significant improvement in VAS scores compared to NHIC patients, with a *P* value of .0056 (Supplementary Figure 6B, Supplemental Digital Content, http://links.lww.com/MD/N590).

**Figure 2.**
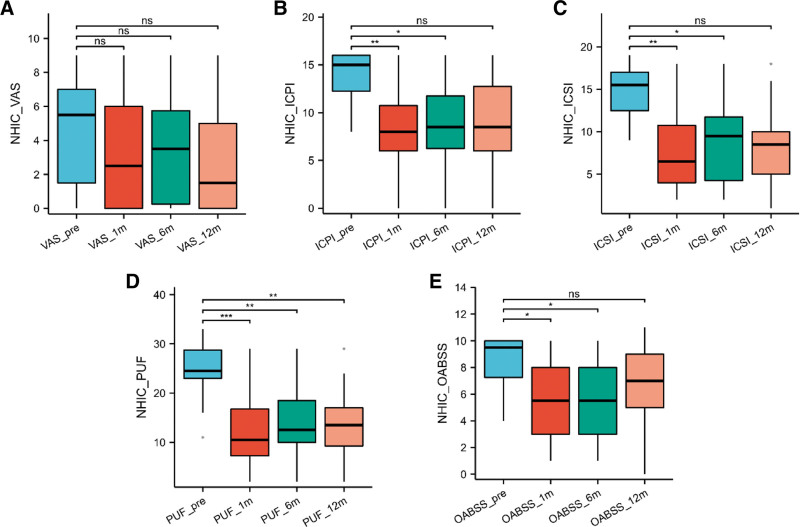
Comparative analysis of follow-up outcomes at 1 yr postoperatively in NHIC patients. (A) Comparative VAS scores at 1 yr postoperatively among different age groups of NHIC patients. (B) ICSI scores at 1 yr postoperatively among different age groups of NHIC patients. (C) ICPI scores at 1 yr postoperatively among different age groups of NHIC patients. (D) Comparative Pelvic Pain and Urgency/Frequency (PUF) Symptom Scale at 1 year postoperatively among different age groups of NHIC patients. (E) Comparative Overactive Bladder Symptom Score (OABSS) at 1 year postoperatively among different age groups of NHIC patients. ICPI = Interstitial Cystitis Problem Index, ICSI = Interstitial Cystitis Symptom Index, NHIC = interstitial cystitis without Hunner lesions, NS = not significant (*P* > .05). *P < .05. **P < .01. ***P < .001.

By considering the follow-up of symptoms at preoperative, 1-, 6-, and 12-month intervals, we conducted non-paired comparisons within each group. As depicted in Figure 2, there was a significant improvement in OABSS scores, ICPI scores, and ICSI scores at 1 and 6 months post-surgery compared to preoperative values (*P* < .05). PUF scores exhibited significant improvement at 1, 6, and 12 months post-surgery compared to preoperative values (*P* < .05).

**Figure 3.**
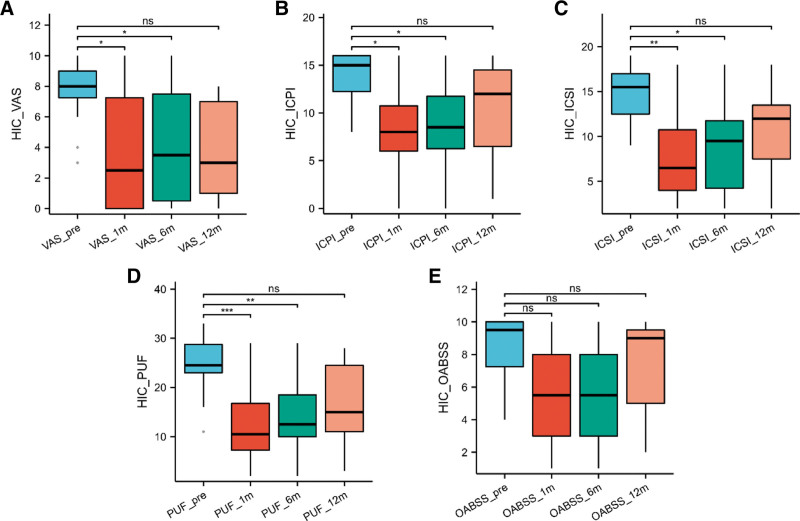
Comparative analysis of follow-up outcomes at 1 yr postoperatively in HIC patients: (A) Comparative VAS scores at 1 yr postoperatively among different age groups of HIC patients. (B) ICSI scores at 1 yr postoperatively among different age groups of HIC patients. (C) Comparative ICPI scores at 1 yr postoperatively among different age groups of HIC patients. (D) Comparative Pelvic Pain and Urgency/Frequency (PUF) Symptom Scale at 1 year postoperatively among different age groups of NHIC patients. (E) Comparative Overactive Bladder Symptom Score (OABSS) at 1 year postoperatively among different age groups of NHIC patients. HIC = interstitial cystitis with Hunner lesions, ICPI = Interstitial Cystitis Problem Index, ICSI = Interstitial Cystitis Symptom Index, NHIC = interstitial cystitis without Hunner lesions, NS = not significant (*P* > .05), VAS = Visual Analog Scale. *P < .05. **P < .01. ***P < .001.

For HIC patients, there was a significant improvement in VAS scores, ICPI scores, ICSI scores, and PUF scores of HIC patients at 1 and 6 months post-surgery compared to preoperative values (*P* < .05) (Fig. 3).

Overall, considering the 1-year follow-up for both NHIC and HIC patients, both groups achieved the lowest scores at 1-month post-surgery, indicating favorable outcomes. However, NHIC patients exhibited a weaker upward trend in scores over time, while HIC patients displayed a gradual increase in scores from the preoperative stage to 1-year post-surgery, and the scores at 1 year after surgery returned to preoperative levels.

## 4. Discussion

Within the cohort of 36 female patients, no statistically significant disparities were observed between individuals classified under NHIC and HIC categories in relation to age, duration of follow-up, frequency of bladder hydrodistention, frequency of bladder perfusion, ICPI, ICSI, PUF, OABSS, maximum detrusor pressure during maximum urinary flow rate, maximum detrusor pressure, bladder compliance, and residual urine volume. However, it is noteworthy that HIC patients exhibited significantly elevated preoperative VAS scores when compared to NHIC patients. Additionally, the HIC group displayed noticeably reduced maximum bladder capacity and anesthesia-induced maximum bladder capacity in comparison to the NHIC group, with these discrepancies reaching statistical significance.

In our study, both cohorts of patients demonstrated substantial and meaningful reductions in symptom scores following bladder hydrodistention, with these improvements persisting over the initial 6-month period. Particularly noteworthy is the fact that HIC patients experienced a more pronounced enhancement in VAS scores at both the 1- and 6-month marks subsequent to CWH, whereas NHIC patients did not.

Analyzing the progression of scores after a 12-month follow-up period, NHIC patients continued to display noteworthy decrease in PUF scores when compared to their preoperative values. Moreover, it is worth mentioning that NHIC patients demonstrated a relatively milder trend of score decrease over time, while HIC patients exhibited a gradual increase in scores from the preoperative stage to 1-year post-procedure, although no significant differences were observed in various scores when compared to preoperative values. There are distinct phenotypic differences between HIC and NHIC patients. The presence of Hunner ulcers observed during cystoscopy may indicate a smaller bladder capacity, and NHIC patients might have a higher tolerance for bladder hydrodistension under anesthesia. HIC patients experience more pronounced pain-related challenges. Therefore, it is reasonable to consider escalating the use of analgesic medications for HIC patients to alleviate pain symptoms and improve their quality of life. CWH surgery leads to more significant improvements in pain symptoms for HIC patients compared to NHIC patients. However, in the long term, HIC patients are more prone to symptom recurrence after 1 year. Therefore, regular bladder perfusion and timely adjustment of treatment plans are necessary post-surgery. NHIC patients tend to maintain treatment effects over a longer period.

Doiron et al^[8]^ conducted at a prominent research center consisting of a cohort of 469 individuals revealed that the interstitial cystitis subtype characterized by Hunner lesions exhibits more severe central bladder symptoms, albeit lacking distinct central bladder phenotypes, they have proposed performing local anesthetic cystoscopy in patients diagnosed with interstitial cystitis/bladder pain syndrome. In addition to identifying Hunner lesions, cystoscopy under local anesthesia allows for the assessment of bladder pain/discomfort during bladder filling and emptying processes, as well as the determination of functional bladder capacity, which may be limited by pain/discomfort. Recent studies have emphasized the importance of CWH in diagnosing interstitial cystitis/bladder pain syndrome (IC/BPS), particularly in identifying Hunner lesions.^[9]^ Cystoscopy under local anesthesia allows for accurate assessment of bladder pain/discomfort and differentiation between HIC and NHIC subtypes.^[10]^ Transurethral fulguration or resection of Hunner lesions remains a highly effective treatment, providing significant symptom relief in over 90% of patients.^[11]^

The phenotypic differences between HIC and NHIC patients underscore the need for individualized treatment strategies.^[12]^ HIC patients often present with more severe nociceptive pain and reduced bladder capacity due to epithelial denudation and chronic inflammation. NHIC patients tend to have milder inflammation and can benefit from regular bladder instillations and CWH.^[13]^ Developing standardized criteria for distinguishing between these subtypes will be crucial for improving diagnostic accuracy and optimizing treatment approaches.

While the conventional belief suggests poor efficacy of standard treatments for chronic IC/BPS, evidence suggests the existence of effective therapeutic approaches for IC/BPS patients with Hunner lesions. Transurethral fulguration of Hunner lesions was first reported by Kerr^[14]^ as early as 1971. Peeker et al^[15]^ reported a study involving 103 patients, wherein the researchers observed improvement in 90% of the cases, with nearly half of the respondents experiencing sustained relief for over 3 years. Although the outcomes of bladder hydrodistention therapy alone have been inconsistent, with symptom improvement rates ranging from 54% to over 90% and variable durations not exceeding 6 to 9 months.^[16–18]^ The American Urological Association guidelines regard cystoscopic hydrodistention as a tertiary treatment modality.^[19]^ However, excision or cauterization of Hunner ulcers has proven highly effective, with 90% of cases experiencing improvement. Some experts recommend that a complete excision can maximize the reduction of bleeding and minimize the risk of bladder contraction.^[15,20]^

Despite the valuable insights gained, this study has several limitations. First, the relatively small sample size and exclusive inclusion of female patients limit the generalizability of the findings. Future studies should consider expanding the cohort size and including male patients to provide a more comprehensive understanding of the disease. Second, the study focused on short-term outcomes following CWH surgery, and longer follow-up periods are necessary to better evaluate the long-term efficacy of the intervention. Additionally, further research should explore the molecular mechanisms underlying the phenotypic differences between HIC and NHIC patients, which may uncover novel therapeutic targets and refine treatment strategies. Moreover, developing standardized criteria for distinguishing between HIC and NHIC subtypes will be crucial in improving the diagnostic accuracy and optimizing individualized treatment approaches. Lastly, comparative studies assessing the efficacy of different treatment modalities in conjunction with CWH surgery will be essential to identify the most effective therapeutic combinations for both HIC and NHIC patients.

In summary, the implementation of CWH surgery emerges as an effective intervention for ameliorating symptoms and mitigating the deleterious effects of pain, nocturia, and other symptoms among HIC and NHIC patients. For HIC patients, once diagnosed, CWH can be performed as soon as possible, and pain can be alleviated as soon as possible by cutting or cauterizing ulcers. Regular follow-up and timely adjustment of treatment plan are also necessary. For NHIC patients, CWH can maintain long-term treatment effectiveness. As a result, this surgical approach holds significant promise for enhancing the overall quality of life experienced by individuals afflicted with IC. These conclusions have guiding significance for clinical diagnosis and treatment.

## Author contributions

**Conceptualization:** Lin Zhu, Kexin Xu.

**Data curation:** Lin Zhu, Hanwei Ke, Qi Wang.

**Formal analysis:** Lin Zhu, Hanwei Ke.

**Investigation:** Lin Zhu, Hanwei Ke.

**Methodology:** Lin Zhu, Hanwei Ke.

**Project administration:** Lin Zhu.

**Supervision:** Lin Zhu, Qi Wang.

**Validation:** Lin Zhu, Hanwei Ke.

**Visualization:** Lin Zhu.

**Writing—original draft:** Lin Zhu, Hanwei Ke.

**Writing—review & editing:** Lin Zhu, Hanwei Ke, Qi Wang, Kexin Xu.

**Funding acquisition:** Kexin Xu.

## Supplementary Material


