# Supplementary material for: Comparative analysis of surgical prognostic between HIC and NHIC patients after cystoscopy with hydrodistention
Source: Medicine (Baltimore). 2024 Sep 20;103(38):e39640. doi: 10.1097/MD.0000000000039640 (PMC11419442; doi:10.1097/MD.0000000000039640)
Supplement: Supplementary file 5 [file medi-103-e39640-s005.docx]

Supplementary Figure 5: Enhancement of Overactive Bladder Symptom Score (OABSS) within 6 Months Postoperatively: Depicts the change in OABSS scores within 6 months postoperatively for all patients.
