# Supplementary material for: Comparative analysis of surgical prognostic between HIC and NHIC patients after cystoscopy with hydrodistention
Source: Medicine (Baltimore). 2024 Sep 20;103(38):e39640. doi: 10.1097/MD.0000000000039640 (PMC11419442; doi:10.1097/MD.0000000000039640)
Supplement: Supplementary file 6 [file medi-103-e39640-s006.docx]

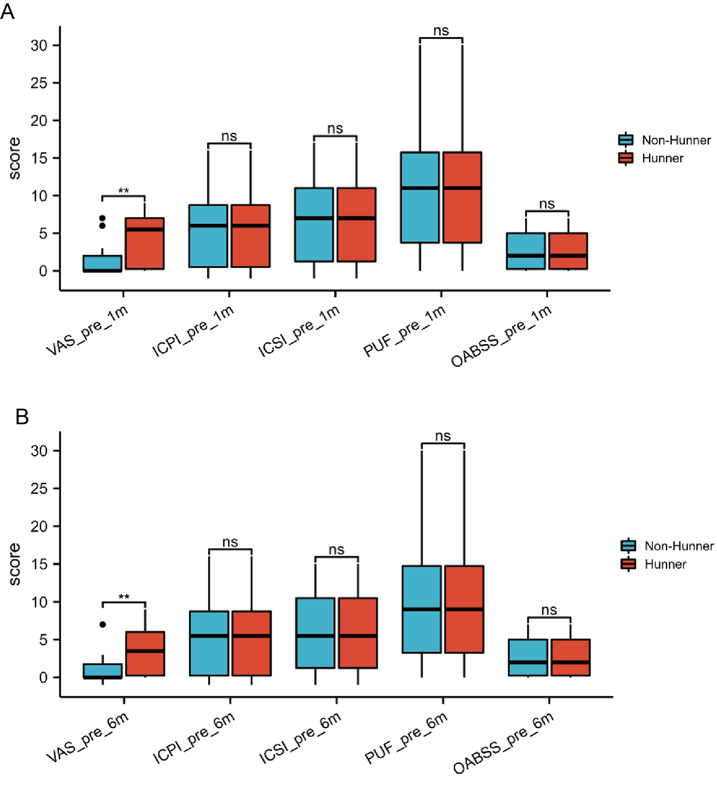


Supplementary Figure 6. Improvement in Visual Analog Scale (VAS) of Both Groups of Patients at 1 and 6 Month Postoperatively Compared to Preoperative Scores: Compares the improvement in VAS scores for both patient groups at 1 and 6 months postoperatively relative to preoperative scores, illustrating changes in pain perception.
